# Network-constrained technique to characterize pathology progression rate in Alzheimer’s disease

**DOI:** 10.1093/braincomms/fcab144

**Published:** 2021-07-15

**Authors:** Fon Powell, Duygu Tosun, Ashish Raj, for the Alzheimer’s Disease Neuroimaging Initiative

**Affiliations:** 1 Department of Radiology, Weill Cornell Medical College of Cornell University, New York, NY 10065, USA; 2 Department of Radiology and Biomedical Imaging, University of California San Francisco, AC-116, Parnassus, Box 0628, San Francisco, CA 94122, USA; 3 San Francisco Veterans Affairs Medical Center, San Francisco, CA 94121, USA

**Keywords:** pathology progression rate, Alzheimer’s disease, network diffusion model, fluid biomarkers

## Abstract

Current methods for measuring the chronic rates of cognitive decline and degeneration in Alzheimer’s disease rely on the sensitivity of longitudinal neuropsychological batteries and clinical neuroimaging, particularly structural magnetic resonance imaging of brain atrophy, either at a global or regional scale. There is particular interest in approaches predictive of future disease progression and clinical outcomes using a single time point. If successful, such approaches could have great impact on differential diagnosis, therapeutic treatment and clinical trial inclusion. Unfortunately, it has proven quite challenging to accurately predict clinical and degeneration progression rates from baseline data. Specifically, a key limitation of the previously proposed approaches for disease progression based on the brain atrophy measures has been the limited incorporation of the knowledge from disease pathology progression models, which suggest a prion-like spread of disease pathology and hence the neurodegeneration. Here, we present a new metric for disease progression rate in Alzheimer that uses only MRI-derived atrophy data yet is able to infer the underlying rate of pathology transmission. This is enabled by imposing a spread process driven by the brain networks using a Network Diffusion Model. We first fit this model to each patient’s longitudinal brain atrophy data defined on a brain network structure to estimate a patient-specific rate of pathology diffusion, called the pathology progression rate. Using machine learning algorithms, we then build a baseline data model and tested this rate metric on data from longitudinal Alzheimer’s Disease Neuroimaging Initiative study including 810 subjects. Our measure of disease progression differed significantly across diagnostic groups as well as between groups with different genetic risk factors. Remarkably, hierarchical clustering revealed 3 distinct clusters based on CSF profiles with >90% accuracy. These pathological clusters exhibit progressive atrophy and clinical impairments that correspond to the proposed rate measure. We demonstrate that a subject’s degeneration speed can be best predicted from baseline neuroimaging volumetrics and fluid biomarkers for subjects in the middle of their degenerative course, which may be a practical, inexpensive screening tool for future prognostic applications.

## Teaser

Powell et al. present a new metric for disease progression rate in Alzheimer’s disease using MRI-derived atrophy data to infer the underlying rate of pathology transmission, via fitting a network diffusion model to patients’ longitudinal brain scans. This rate metric was shown clinically and pathologically relevant in a large Alzheimer cohort.

## Graphical Abstract

**Graphical Abstract:**
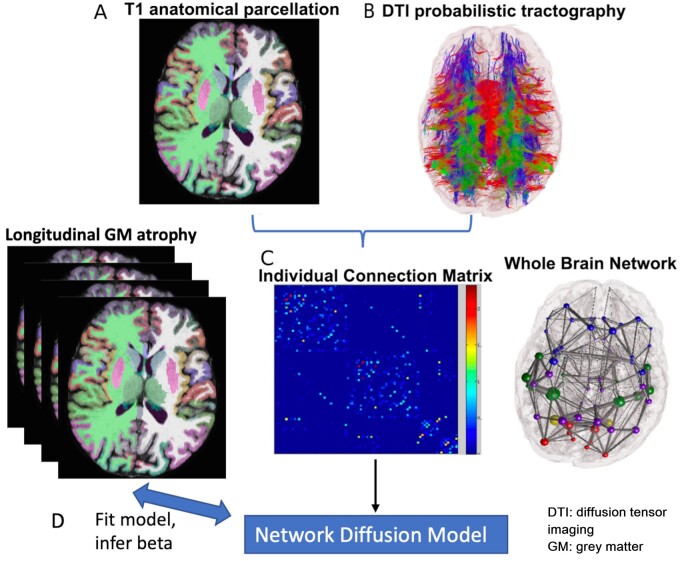

## Introduction

Pathological processes in the brains of Alzheimer’s disease patients start well before clinical symptoms^1^^,^^2^ and give rise to a long and stereotyped progression of regional pathology, atrophy and synaptic dysfunction. The possibility of capturing and predicting longitudinal decline and clinical outcomes from baseline data is a matter of intense interest, with applications in differential diagnosis, therapeutic treatment and clinical trial inclusion.^3–6^ Current measures of decline rely on changes in global clinical assessments such as Mini-Mental State Exam (MMSE) and ADAS-Cog, and longitudinal changes in global brain volume.^7^^,^^8^ However, these measures have proved challenging for the purpose of monitoring and predicting rates of decline.^9^^,^^10^

As a measure of disease progression, global brain volume is limited, as it ignores the highly specific spatial patterns of Alzheimer’s disease-related atrophy. While it is of course possible to obtain atrophy rates for each brain region, in clinical settings a single rate metric is more favourable for prognosis or as primary endpoint in clinical trials. Furthermore, the global atrophy rate does not capture the Alzheimer’s disease-specific progression process intrinsically involving network-level spread, a well-studied ‘prion-like’ transsynaptic transmission of neurodegenerative processes.^11–13^

To address these limitations, this paper presents a new measure of pathology progression rate (PPR) in Alzheimer’s disease-spectrum patients, using at its core a Network Diffusion Model (NDM) that was previously shown to recapitulate spatial and temporal patterns of Alzheimer’s disease-related neurodegeneration spread along with the brain’s white matter fibre architecture.^14^ The strength of this approach is that it connects a biophysically realistic model of protein transmission to the whole brain regional level. Therefore, we propose the NDM as a robust model from which a PPR metric can be derived directly from MRI-based atrophy maps.

The current work makes three main contributions: First, we propose an algorithm to compute the PPR from longitudinal MRI of a subject, by fitting the NDM’s (*a priori* unknown) rate parameter called the diffusivity rate β, as a primary metric of PPR. Although the NDM has been extensively used in various dementias,^15–23^ it has previously focussed on cross-sectional rather than longitudinal data. The PPR proposed here encapsulates the entire spatiotemporal evolution of the disease and serves as a mechanistically relevant measure of rate of progression in the Alzheimer’s disease spectrum. Second, we thoroughly characterize the PPR parameter on a large cohort of 810 subjects across the Alzheimer’s disease spectrum available in the Alzheimer’s Disease Neuroimaging Initiative (ADNI) study.^24^ Specifically, we assess to what extent the PPR parameter is explained by the well-established phenotypical (i.e. clinical diagnosis) and genotypical (i.e. ApoE genotype) factors. Third, we assess the predictive value of baseline Alzheimer’s disease biomarkers including atrophy measures from MRI and CSF Aβ and tau levels for subject-specific PPR metric. A previous approach dealt with longitudinal fitting of NDM^25^ but did not attempt to predict it from baseline. To our knowledge, this is the first study to propose a network-based disease progression rate metric built on a known mechanism of disease pathology progression in Alzheimer’s disease.

## Methods

### Participants

All subject data were obtained from the public longitudinal Alzheimer’s Disease Neuroimaging Initiative (ADNI) database (http://adni.loni.usc.edu/). Subjects were diagnosed as Alzheimer’s disease, Late Mild Cognitive Impairment (LMCI), Early Mild Cognitive Impairment (EMCI) or Control (CON) according to published ADNI diagnostic criteria. Only subjects who had two or more longitudinal volumetric MRI were included in this study. Subjects in different diagnostic groups were age- (Alzheimer’s disease: *P* = 0.65, LMCI: *P* = 0.77, EMCI: *P* = 0.10) and gender-matched (Alzheimer’s disease, *P* = 0.06, LMCI: *P* = 0.76, EMCI: *P* = 0.77) healthy controls. Forty CON subjects were eliminated to age-match for LMCI and EMCI subjects. Individuals who had diagnosis conversion (e.g. CN to MCI, MCI to Alzheimer’s disease) were excluded to ensure the integrity of subsequent clustering analysis.

### Structural MR image acquisition and processing

ADNI gives 3 T structural T_1_-MRI (3D Magnetization Prepared Rapid Acquisition Gradient Echo or Inversion Recovery—Spoiled Gradient Echo) with sagittal slices and voxel size of 1 × 1.2 × 1.2 mm^3^ (http://adni.loni.usc.edu/methods/documents/mri-protocols). Automated cortical and subcortical volume measures were performed with FreeSurfer software, version 5.3, using their longitudinal processing stream (http://surfer.nmr.mgh.harvard.edu/fswiki). All imaging protocols and preprocessing procedures are available on the ADNI website. (http://adni.loni.usc.edu/methods/).

### DWI acquisition and processing

The white matter connectome was constructed from MRI data of 73 (40 men, 33 women, 30.2 ± 6.7 years) normal, healthy subjects collected previously.^26^^,^^27^ T_1_-weighted structural and diffusion-weighted MR images were collected on a 3 T GE Sigma EXCITE scanner. High Angular Resolution Diffusion Imaging scans were acquired with 55 isotropically distributed diffusion-encoding directions at *b *=* *1000 sec/mm^2^ and one at *b *=* *0 sec/mm^2^, from 72 1.8-mm thick interleaved slices (no slice gap) and 128 × 128 matrix size, zero-filled during reconstruction to 256 × 256, with a field of view of 230 mm^2^. T_1_-weighted inversion-recovery fast spoiled gradient-recalled echo sequence (Echo time = 1.5 msec, Repetition time = 6.3 msec, Inversion time = 400 msec) flip angle of 15°) with a 256 × 256 matrix over a 230 mm^2^ field of view and 156 1.0-mm contiguous partitions.

High Angular Resolution Diffusion Imaging was corrected for eddy current and motion artefacts using FMRIB Software Library (FSL).^28^ T_1_ images were processed by first segmenting the tissue into CSF, white matter (WM) and grey matter (GM). The GM segment was subsequently parcellated into 86 different ROIs of the FreeSurfer (Desikan-Killiany) parcellation corresponding to 68 cortical and 18 subcortical structures. The parcellated GM was then linearly transformed and resampled to diffusion image space for use in tractography.

### Tractography and anatomic connectivity graph

**Figure 1:**
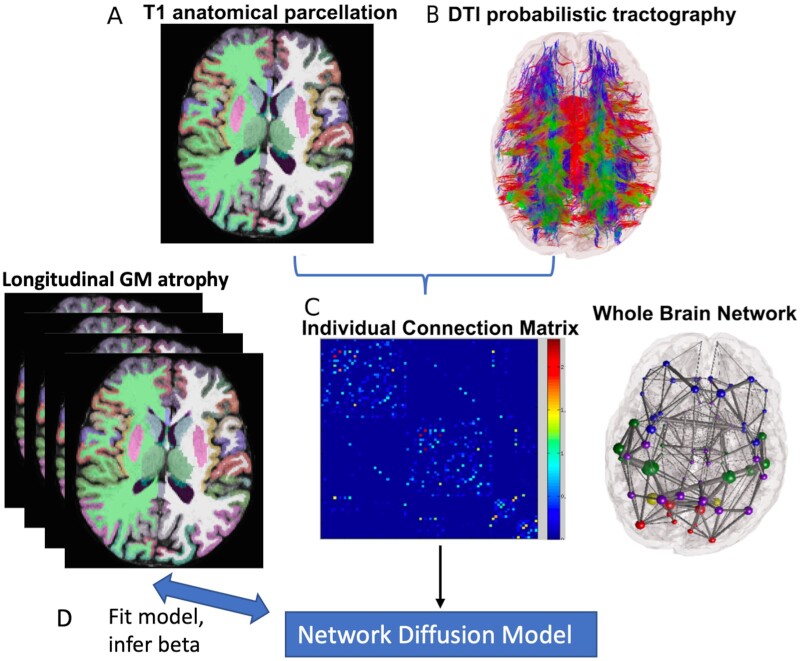
**Analysis pipeline to yield PPR.** Using an atlas parcellation, here, Desikan-Killiany 86-region atlas (**A**) and probabilistic tractography on diffusion tensor imaging (DTI) (**B**) a brain connectome was created (**C**), with expected sparsity and topology. The NDM was evaluated on this connectome, and its beta parameter was fitted in order to produce the maximum match with the subject’s empirical longitudinal atrophy (**D**). The fitted beta is henceforth also referred to as the pathology progression rate (PPR).

High Angular Resolution Diffusion Imaging data were processed using spherical deconvolution to yield orientation distribution functions in each voxel. The resulting output was fed into a probabilistic fibre tracking algorithm, to produce fibre tracts in terms of streamlines (see Fig. 1). Each voxel at each region’s grey–white interface was seeded with 100 streamlines and the resulting tracts were traced probabilistically. Proposed and validated in^29^ the tractography algorithm implemented here incorporates tissue classification probability and orientation distribution information in a Bayesian manner. A tract terminated when the algorithm reached the boundary of an image volume, the edge of a GM region, a voxel not in the grey or WM masks, or when the angle between subsequent steps exceeded π/3. This analysis was done using Statistical Parametric Mapping (SPMv8),^30^ a software package within Matlab (The Mathworks, Inc., Natick, MA), and the Individual-Based Atlas toolbox^31^ within Statistical Parametric Mapping.

The number of streamlines (weighted by probability score) found to go from region i to region j and vice versa, were averaged and this value was recorded as the connectivity $c_{i,j}$ between the two regions. The full 86 × 86 connectivity matrix was then formed as per $C=\{c_{i,j}\}$. Connectivity matrices from individual healthy subjects were found to be largely similar, nonetheless, to control for inter-subject variance in total fibre count, each healthy subject’s connectome was normalized by their total (weighted) number of streamlines. Then a mean over all subjects was taken in order to obtain a canonical healthy connectivity matrix C, which was used in all subsequent analysis. The subsequent undirected canonical connectome was weighted, not binary. The weights were minimally thresholded to remove very small entries, which yielded ∼85% sparsity—as depicted in the example in Fig. 1. Further details of the image processing and tractography method are given in a previous publication.^26^^,^^27^

### The Network Diffusion Model

Our group’s previously published NDM has been shown to accurately recapitulate spread of Alzheimer’s Disease pathology throughout the brain.^14^^,^^25^ Briefly, pathology transmission is modelled as a network diffusion process:

(1) $$x\left(t\right)=\;e^{-\beta Ht}x_{0}$$

where $x_{0}$ and x(t) are the initial (*t* = 0) and evolved patterns of the regional pathology at time t respectively, expressed as 86×1 vectors denoted in boldface, corresponding to the 86-region brain atlas. The matrix exponential exp ⁡(-βHt) acts as a spatial and temporal blurring operator, called the diffusion kernel. Matrix H, called the network Laplacian, is a derivative of the connectivity matrix obtained from the white matter structural organization. The parameter β represents the diffusion rate coefficient, which is considered the rate of disease progression (i.e. speed of neurodegeneration) in the current study.

**Relationship to atrophy**. The measurable phenotype (regional MRI-derived atrophy) in region k is assumed to be the consequence of and thus proportional to the *accumulation* of pathology. Hence, it is modelled as the integral:

(2) $$\phi_{k}\left(t\right)=\int_{0}^{t}x_{k}\left(\tau \right)\;d\tau$$

On the whole brain this gives:

(3) $$\Phi \left(t\right)=\;\int_{0}^{t}x\left(\tau \right)\;d\tau$$

***Relationship between baseline atrophy and its rate of change.*** Let $\Phi_{\mathrm{base}}$ be the baseline atrophy pattern vector of a given subject and let $t_{\mathrm{base}}$ be the (unknown) time elapsed between pathology initiation and the baseline exam. It was shown in Raj et al.^14^^,^^25^ that the relationship at baseline scan between atrophy and its rate of change (as modelled by NDM) under the integral relationship above is given by

(4) $$\frac{d\Phi }{dt}{|}_{t=t_{\mathrm{base}}}\;=\;\beta \tilde{H}\left(\beta t_{\mathrm{base}}\right)\Phi_{\mathrm{base}}$$

where we define a new variant of the Laplacian

(5) $$\tilde{H}\left(\beta t\right)=U\;\mathrm{diag}\left(\left\{\begin{array}{cc} \frac{1}{\beta t},\; & i=1\\ \frac{\lambda_{i}e^{-\lambda_{i}\beta t}}{{1-\;e}^{-\lambda_{i}\beta t}},\; & i>1 \end{array}\;\right\}\;\right)U^{†}$$

involving the eigendecomposition $H=U\Lambda U^{†}$, with eigenvalues $\lambda_{i}$. Please see Raj et al.^25^ for details. The new term $\tilde{H}$ is not a conventionally defined Laplacian, but it is the correct operator for derivatives of a signal that satisfies Equations (1) and (3). Thus, the NDM deterministically predicts that the atrophy at baseline and its rate of change are related via the matrix $\tilde{H}(\beta t)$.

Please note that pathology modelled by the NDM can decrease with time, especially in seeding sites, since the NDM is a mass-conserving model. However, we do not have direct access to pathology in this study, only atrophy. By assumption, atrophy is the integral of pathology (Equation (3)), hence the former can never go down, even if the latter does. Note also that processes like hypertrophy or compensatory mechanisms that can in some cases cause decrease in atrophy are outside the scope of the present model.

### Algorithm for fitting β to individual subjects’ longitudinal scans

For each subject, the β parameter is computed using the subject’s longitudinal atrophy patterns. An illustration of the analysis pipeline is given in Fig. 1.

The algorithm has the following steps:

1. Atrophy slope of each region is calculated independently, using $t_{\mathrm{long}}$, a vector of timestamps (length *nt*) of a patient’s longitudinal visits. Let the regional atrophy patterns be contained in the (*nt x 1*) vector for any given region r, $\phi_{r}$. A fitted estimate of slope of atrophy for that region is calculated from this longitudinal data, using linear regression with intercept. Mathematically:

(6) $$\phi_{r}=\;\left(1\;t_{\mathrm{long}}\;\right)\left(a_{r}\;b_{r}\right)$$

where 1 is a *nt x 1* vector of ones and $t_{\mathrm{long}}$ is a *nt x 1* vector of longitudinal time elapsed since baseline (in years). Hence the fitted coefficient $a_{r}$ gives the intercept and $b_{r}$ the slope of empirical atrophy, which we record as $\frac{\Delta \phi_{r}}{\Delta t}=b_{r}$. We collect all fitted regional slopes in the vector $\frac{\Delta \Phi }{\Delta t}=\{\frac{\Delta \phi_{r}}{\Delta t}|\forall r\}$. In our implementation, the above fitting was performed using MATLAB’s pseudoinverse function. Regions with negative atrophy slopes are set to 0, as thickening of the cortex is a rare and unlikely event in Alzheimer’s disease, and most likely is a result of measurement error.

Note that the relationship between instantaneous regional atrophy and its instantaneous slope in Equation (4) is exact, not a linearized assumption. However, the deduction of the empirical derivative via Equation (6) requires non-instantaneous (longitudinal) data. Hence there is an implicit assumption of linear change within the longitudinal time window of 2–6 years. In practice, the Alzheimer’s disease course runs into decades, hence the purpose of computing empirical atrophy slopes the implicit linear assumption is realistic.

To estimate β and $t_{\mathrm{base}}$ parameters jointly a two-step approach as detailed in steps 2 and steps 3 was used:

2. Set $\beta =\beta_{0}$, where $\beta_{0}$ is an initial or default value, set as $0.05\;{\mathrm{year}}^{-1}$. Note that the unit of PPR is 1/time. Hence $0.05\;{\mathrm{year}}^{-1}$ represents a rate of progression that should correspond to 0.05 per year. Then minimize over $t_{\mathrm{base}}$:

(7) $$\hat{t_{\mathrm{base}}}=\;\mathrm{argma}x_{t_{\mathrm{base}}}\;\{\mathrm{corr}(\beta_{0}\tilde{H}\left(\beta_{0}t_{\mathrm{base}}\right)\Phi_{\mathrm{base}},\frac{\Delta \Phi }{\Delta t})\}$$

where corr(⋅,⋅) refers to the Pearson correlation coefficient between two vectors, and recall that $\frac{\Delta \Phi }{\Delta t}$ is the measured slope from step 1.

3. Then the optimal rate $\hat{\beta }$ is given by least squares fitting, obtained via MATLAB’s pseudoinverse, of β in the linear system:

(8) $$\frac{\Delta \Phi }{\Delta t}=\beta \tilde{H}\left(\beta_{0}\hat{t_{\mathrm{base}}}\right)\Phi_{\mathrm{base}}$$

Although steps 2 and 3 can in theory be iterated, in practice it was found sufficient to perform this process once only.

### Statistical analysis

Following calculation of each subject’s β parameter from baseline and subsequent longitudinal volumetric scans, β outliers were removed. Outliers are defined as 1.5 times greater or less than the inter-quartile range separately for each diagnostic group. This was necessitated by the observation that the key measurement in its estimation is the derivative $\frac{\Delta \Phi }{\Delta t}$, which is very noisy. Note that above analysis requires only baseline and slope, allowing us to use subjects with multiple and varying number of visits. It is likely that the quality of the slope estimate will be better for >2 time points, but we did not explore this effect in this study.

Differences in subject characteristics across diagnostic groups, APOE status categories and clusters were assessed with the Kruskal–Wallis H-test, a non-parametric one-way ANOVA on ranks.^32^ Wilcoxon rank-sum tests were used in the case of comparing 2 groups directly.^33^ Differences in frequencies were assessed with classic chi square analysis. Independent, two-sided *t*-tests were used to test for differences in local brain volume. Glass brains depicting magnitude and t-statistic are used for visualization.^34^ Multivariate linear regression analysis was used to examine the relationship between multiple CSF biomarkers, cortical/subcortical volumes and β. The coefficient of determination, *R*^2^ was used to evaluate model fit.

*Z*-scores of CSF metrics were computed relative to age-matched controls to normalize features for hierarchical clustering analysis. Hierarchical clustering analysis was used to delineate clusters of subjects in a six-dimensional space defined by baseline CSF biomarkers of Aβ, Tau, phosphorylated tau (pTau) and their respective ratios. The optimum cluster size was estimated based on the variance ratio criterion (Calinski–Harabasz index), defined as the ratio of between-cluster variance to within-cluster variance is maximized at 3 clusters.^35^ Four hundred ninety patients on the Alzheimer’s disease spectrum of 578 patients (excluding outliers) had complete data on all 6 CSF biomarkers of interest in the current study and were used in clustering analysis. All analysis was carried out in MATLAB.

### Data availability

All data used in this study are pubic, from the ADNI database available from (http://adni.loni.usc.edu/). Computer code will be available freely on our GitHub repository (https://github.com/Raj-Lab-UCSF).

## Results

Subject demographic information is provided in Table 1. 92% of subjects have baseline CSF biomarkers. All subjects also had measures of the MMSE and Functional Activities Questionnaire (FAQ) to assess global clinical/cognitive impairment. Since 99% of subjects’ MMSE and FAQ scores were collected within 1.25 years of their baseline MRI (mean = 0.59 years, median = 0.55 years), we consider these scores as baseline measures. Four EMCI subjects had baseline cognitive metrics measured ∼2 years post-baseline scan.

**Table 1:** ADNI subject demographics

Dx	*N*	Age (std)	Sex (% F)	APOE4 status (0/1/2)	MMSE (std)	FAQ (std)	CSF AB (pg/ml) (std)	CSF Tau (pg/ml) (std)	CSF pTau (pg/ml) (std)
Alzheimer’s disease	117	74.9	38%	24/51/22	21.6	15.5	134.6	131.2	58.9
		(8.1)		83%+	(3.9)	(6.9)	(37.2)	(64.8)	(32.9)
					85%+	84%+	94%+	89%+	94%+
LMCI	172	72.5	45%	74/69/27	26.7	4.8	159.4	99.8	47.6
		(7.7)		99%+	(2.4)	(5.7)	(49)	(54.5)	(27.4)
					97%+	97%+	94%+	89%+	94%+
EMCI	289	71.3	44%	161/103/20	28	2.3	183.8	77.2	36.8
		(7.2)		98%+	(1.8)	(3.5)	(51.2)	(47.6)	(21.1)
					95%+	95%+	94%+	89%+	94%+
CON	232	74.5	53%	153/56/6	28.9	0.44	198.1	68	33.1
		(6.9)		93%+	(1.3)	(2)	(51.2)	(33.7)	(17.9)
					91%+	90%+	94%+	89%+	89%+

APOE, apolipoprotein-E; CON, healthy controls; EMCI, Early Mild Cognitive Impairment; FAQ, Functional Activities Questionnaire; LMCI, Late Mild Cognitive Impairment; MMSE, Mini-Mental State Exam.

+Denotes percentage of data available in diagnostic cohort.

**Figure 2:**
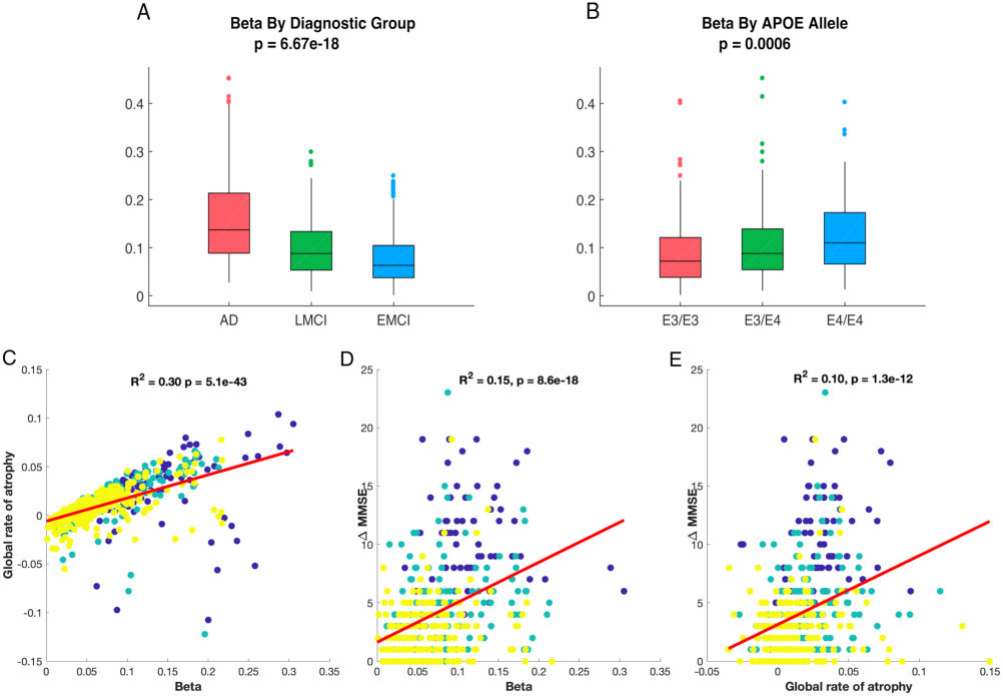
**Significant effect of PPR on subject’s atrophy rate, MMSE, diagnosis and genetics.** (**A**) The fitted value of beta, or PPR, is significantly higher in Alzheimer’s disease than MCI groups. (**B**) Fitted beta is significantly higher in subjects with E4/E4 APOE alleles compared to other APOE groups, across the Alzheimer’s disease spectrum. Subjects with both putative APOE4 alleles show the highest betas, while subjects homozygous for the APOE3 allele exhibit the lowest betas. (**C**) Correlation between a subject’s fitted PPR and global atrophy rate. (**D**) Correlation between each subject’s β and slope of clinical decline as measured by the difference between end-point MMSE and unimpaired MMSE (i.e. 30). Notice inverted scale. Blue represents Alzheimer’s disease, teal represents LMCI and yellow represents EMCI. (**E**) For comparison the correlation between clinical decline and global rate of atrophy is shown. While both **D** and **E** give moderate association, the one using beta is stronger.

We test whether the network-derived beta parameter that characterizes the PPR varies by the subject’s diagnostic and genetic status. Prion hypotheses in Alzheimer’s disease suggest the disease spreads in an exponential, viral-like manner, with the disease spreading faster with increased disease severity.^13^ Thus, we hypothesized that subjects later in clinical progression will have a higher rate of disease spread than subjects early in the disease. Although we accommodate APOE status and CSF biomarker levels in our analysis, we did not specifically stratify our subjects based on amyloid positivity, under the emerging concept that amyloid facilitates rather than governs the spatiotemporal trajectory of Alzheimer’s disease spectrum subjects. To test the hypothesis, each subject’s PPR (i.e. β) was calculated and its effect across each of the three diagnostic groups was assessed using a non-parametric Kruskal–Wallis H test. We observe a significant effect of β across diagnoses on the Alzheimer’s disease spectrum (*P* = 6.67e-18). Furthermore, when directly comparing the earliest stage EMCI group to the Alzheimer’s disease group, we show a significant difference in β between EMCI and Alzheimer’s disease patients (*P* = 5.72e-18) (Fig. 2A). Note, healthy subjects were used to compute statistically normalized effect sizes in patients but are themselves not included here as a separate cohort.

Since Alzheimer’s disease is highly associated with the number of putative APOE4 alleles, we examined the relationship between β and APOE4 allele status.^36^^,^^37^ In our dataset, 266 patients have 0 APOE4 alleles, 195 patients have 1 allele and 66 have 2 APOE4 alleles. Thus, we hypothesized that β is higher in subjects with the pathogenic APOE4 allele compared to subjects without the APOE4 allele. A one-way Kruskal–Wallis test confirms this, illustrating a significant effect of beta across subjects with 0, 1 and 2 APOE4 alleles (*P* = 0.0006) Specifically, we observe beta in subjects with the E4/E4 allele is significantly higher than subjects without the E4 allele (*P* = 7.50e-04) (Fig. 2B).

Now we establish whether PPR is reflective of longitudinal progression of neurodegeneration. We hypothesize that there will be a strong relationship between beta and the global slope of atrophy as well as a strong relationship between beta and clinical/cognitive decline measured via MMSE. Indeed, a high correlation was found between the modelled beta parameter and global rate of atrophy ($R^{2}=0.30,\;P=5.1\times 10^{-43}$) (Fig. 2C). A moderate correlation was found between the modelled beta parameter and clinical decline as measured by the difference between unimpaired MMSE (i.e. 30) and subject’s end-point MMSE ($R^{2}=0.15,\;P=8.6\times 10^{-18}$) (Fig. 2D). For comparison, we also obtained the correlation of clinical decline with global slope of atrophy (Fig. 2E), which, while moderately significant ($R^{2}=0.10,\;P=1.3\times 10^{-12}$), was weaker compared to that of beta. These *P*-values were not corrected for multiple comparisons. The patient subgroups showed no discernible differences in *R*, hence were not separately evaluated; although clearly Alzheimer’s disease give the highest and EMCI the lowest average values of both global atrophy rate and fitted β.

Since the NDM model in this study was evolved on the Cornell connectome from prior publications,^26^^,^^27^ we wished to assess whether using a different, more modern connectome would also produce similar results. To test this, we employed the average connectome from the Human Connectome Project, see e.g. Fornari et al.^38^ We found similar, although slightly less strong, results to those shown in Fig. 2; see Supplementary Fig. 1.

We further tested the notion that clinical trajectories of subjects with higher baseline pathology burden worsen faster than subjects with lower burden.^6^^,^^39^ In this study, we accommodated amyloid burden from CSF rather than from PET imaging. We individually probed baseline CSF amyloid beta, tau, ptau, ratio of AB/tau, AB/pTau and pTau/tau. We hypothesize a significant relationship between at least one of baseline CSF biomarkers and speed of pathology spread, both within all subjects and within individually diagnostic groups.

**Figure 3:**
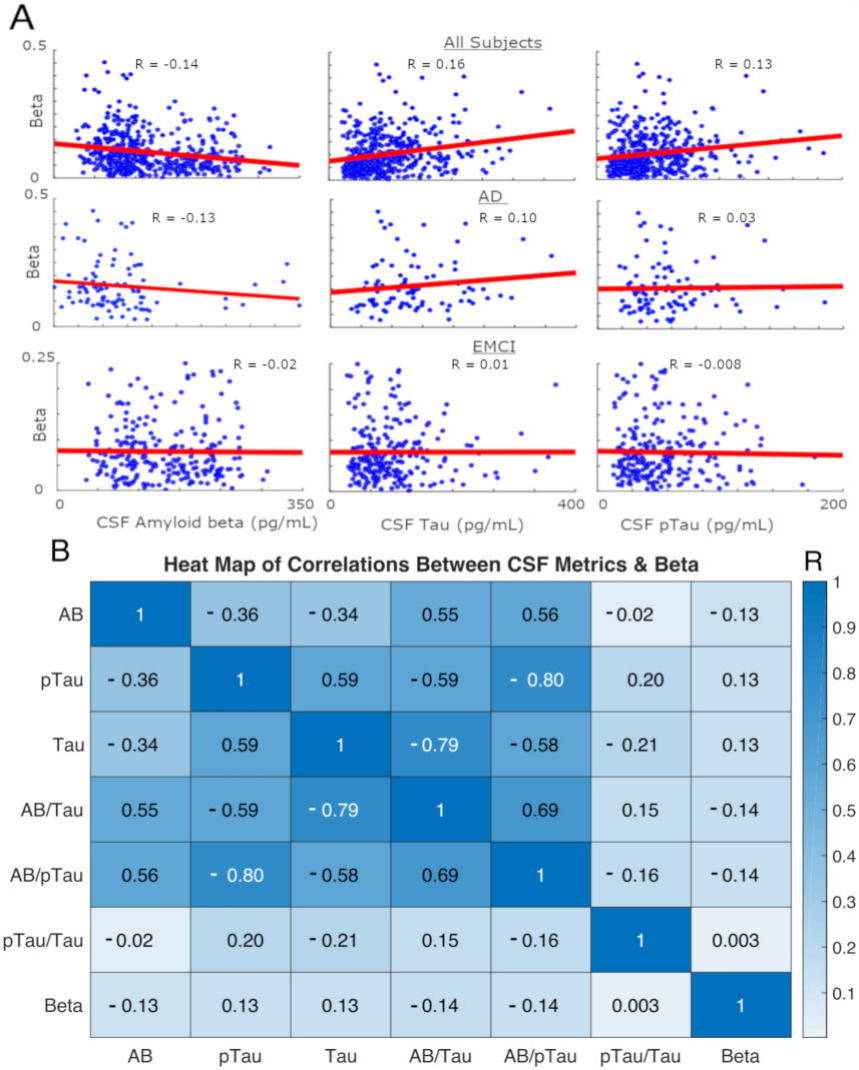
**Correlation analysis between baseline CSF biomarkers and speed of pathology spread.** (**A**) Scatterplots depicting lack of strong correlations between baseline CSF AB, Tau, pTau and their ratios versus beta in all subjects, Alzheimer’s disease-subjects only and EMCI-subjects only. (**B**) Heat map illustrating correlations of baseline CSF measurements with one another and beta.

Contrary to our hypothesis, correlation analysis reveals weak relationships between β and baseline CSF measurements (Fig. 3A). Though baseline CSF values are highly correlated with one another, correlation to β is overall poor (Fig. 3B). Additional linear, quadratic and power models did not reveal significant relationships between CSF biomarkers and beta (data not shown). Multivariate linear regression using multiple CSF biomarkers as predictor variables for β also yielded poor predictive ability as assessed by the coefficient of determination (data not shown).

**Figure 4:**
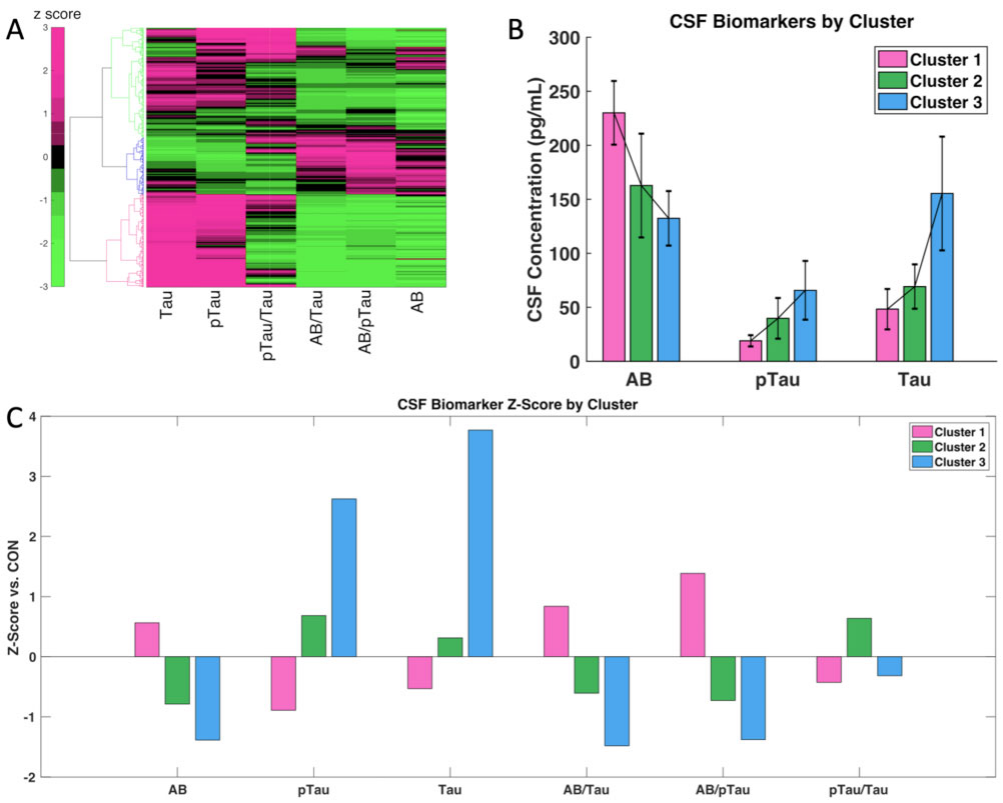
**Clustering and prediction based on CSF biomarker profile.** (**A**) Dendrogram depicting 3 clusters defined by *z*-scores of baseline CSF biomarkers. (**B**) CSF metrics across clusters. Error bars represent one standard deviation. (**C**) Average *z*-score of CSF metrics versus age-matched controls.

Since our efforts to predict a patient’s precise β from baseline CSF measurements were unsuccessful, we hypothesized this was due to potential subject heterogeneity in CSF profile. There is precedent for this hypothesis, as prior work shows CSF biomarker profile across the Alzheimer’s disease spectrum is variable, with clusters of subjects.^40^ Hierarchical clustering analysis was utilized to understand potential subject heterogeneity in baseline CSF biomarker profile. As shown in Fig. 4, 3 clusters are clearly delineated by the *z*-scores of 6 baseline CSF biomarkers: Cluster 1 (*n* = 108, mean age: 71.3 years), Cluster 2 (*n* = 207, 72.9 years) and Cluster 3 (*n* = 175, 72.7 years). There is no significant effect of age across clusters (*P* = 0.20). The average biomarker values in each cluster are shown in panel B for 3 chief CSF biomarkers. For reference the *z*-scores of the 6 CSF biomarker combinations computed relative to age-matched, healthy controls are plotted for each cluster in panel C. Cluster 1 was characterized by the least pathogenic biomarker profile whose difference relative to controls was very small across all 6 biomarkers. In contrast, Cluster 3 appears to be the most pathogenic cluster, showing the most remarkable differences in CSF biomarkers compared to controls. This cluster mirrors the pathogenic biomarker signature of typical of Alzheimer’s disease, which includes decreased CSF AB and increased Tau and pTau compared to controls. Cluster 2 falls between Clusters 1 and 3 in terms of pathogenic profile. Compared directly to each other, Cluster 3 appears significantly more pathogenic than Cluster 1, exhibiting significantly lower baseline CSF amyloid beta compared to Cluster 1 and significantly higher pTau and Tau (AB: *P* = 1.9e-43, pTau: *P* = 5.6e-45, Tau: *P* = 1.8e-44).

**Figure 5:**
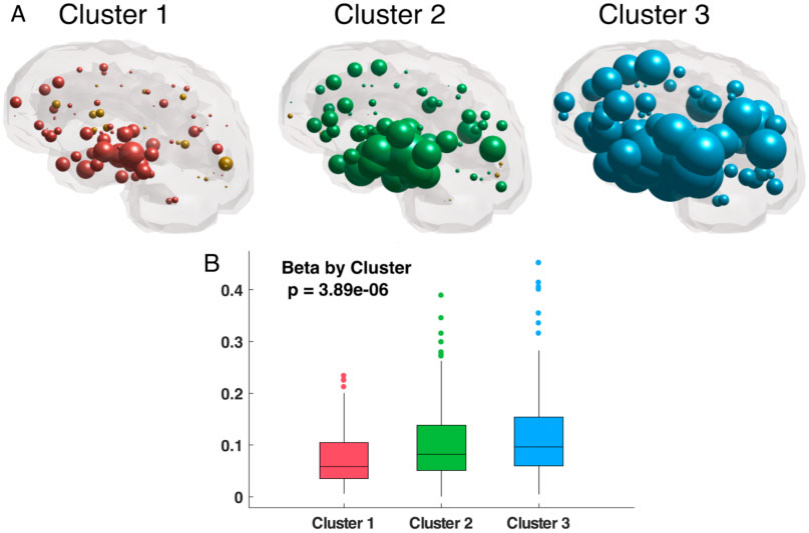
**Baseline volumetric and degeneration speed by cluster.** (**A**) Glass brains depict *t*-statistics of differences in baseline volumetric by cluster compared to age-matched controls Gold represents hyperatrophy, all other colours represent atrophy. (**B**) Boxplot of beta by cluster.

Having successfully clustered subjects based on CSF biomarker profiles, we examine whether these clusters also show significant differences in their baseline volumetrics and in their inferred PPR (Fig. 5). We observed that baseline atrophy patterns correspond to baseline CSF profiles. Specifically, Cluster 3 is characterized by the most amount of atrophy, while Cluster 1 by the least. Cluster 2 falls squarely in the middle. The same is true of inferred PPR parameter β: there is a significant effect of β across clusters (*P* = 3.9e-6). Specifically, the cluster defined by the most pathogenic Alzheimer’s disease-related CSF biomarkers, Cluster 3, shows the highest PPR. In contrast, cluster 1, which is characterized by the least pathogenic biomarkers, has the lowest PPR.

**Figure 6:**
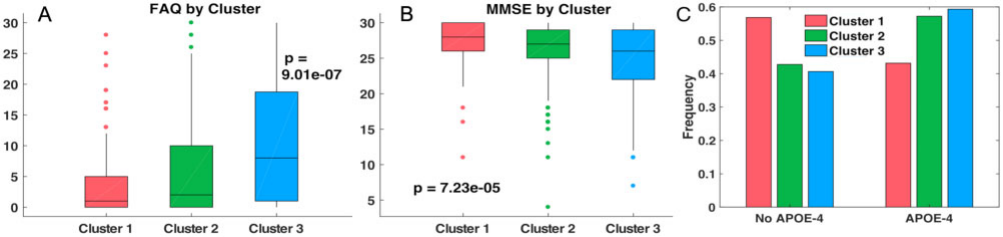
**MMSE, FAQ and APOE differences across clusters.** (**A–B**) Differences in baseline cognitive score FAQ and MMSE across cluster. (**C**) Frequencies in APOE4 across clusters. Pink represents cluster 1, green represents cluster 2 and blue represents cluster 3.

We also observed a significant effect of baseline FAQ and baseline MMSE scores across clusters (FAQ: *P* = 9.0e-7; MMSE: 7.2e-5, Fig. 6). Clinical measures are most compromised in Cluster 3 versus Cluster 1 (FAQ: *P* = 8.0e-07, MMSE: *P* = 1.7e-05), a finding that converges with each cluster’s biomarker profile and rate of disease spread. Correspondingly, we observed that the more pathogenic Cluster 3 has a higher, but non-significant, percentage of APOE4 allele carriers compared to the least pathogenic cluster.

**Figure 7:**
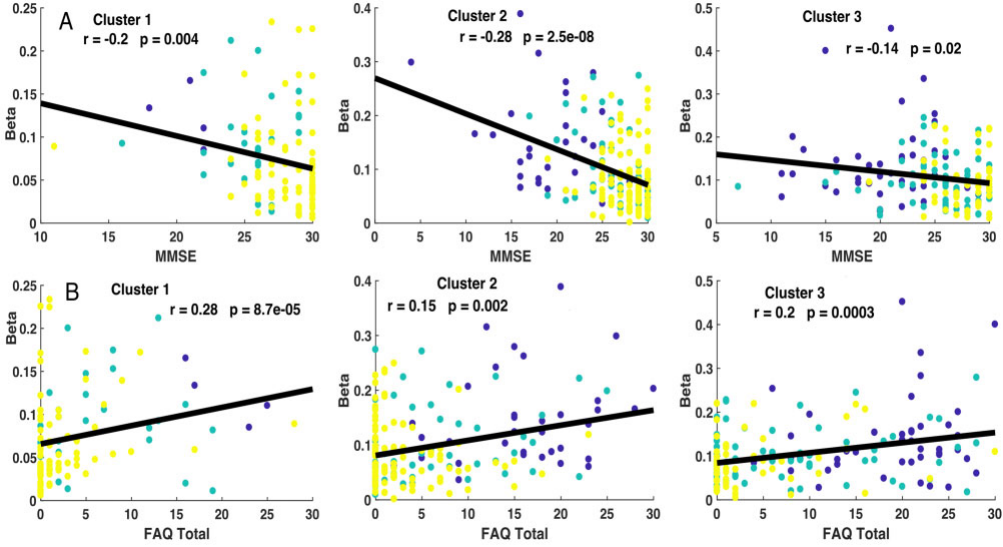
**Correlation between beta and baseline cognitive score within clusters.** (**A**) Correlation between rate of disease progression and baseline MMSE cognitive score across each of the three clusters. (**B**) Correlation between beta and baseline FAQ cognitive score across each of the three clusters. Blue represents Alzheimer’s disease, turquoise represents LMCI and yellow represents EMCI. *P*-values are uncorrected for multiple comparisons. Please note, the *y*-axis on the subplots are not identical.

Lastly, we found correlations between a subject’s speed of disease spread PPR and baseline cognitive score, which differ across clusters (Fig. 7). Cluster 2 had the strongest relationship between β and the baseline MMSE (*r* = −0.28, *P* = 2.5e-8), followed by Cluster 1 (*r* = 0.20, *P* = 0.004) and a relatively weak Cluster 3 (*r* = −0.14, *P* = 0.02). Interestingly, the FAQ survey shows the strong relationship with β in Cluster 1 (*r* = 0.28, *P* = 8.7e-05), followed by Cluster 3 (*r* = 0.20, *P* = 0.0003) and is the weakest in Cluster 2 (*r* = 0.15, *P* = 0.002).

**Figure 8:**
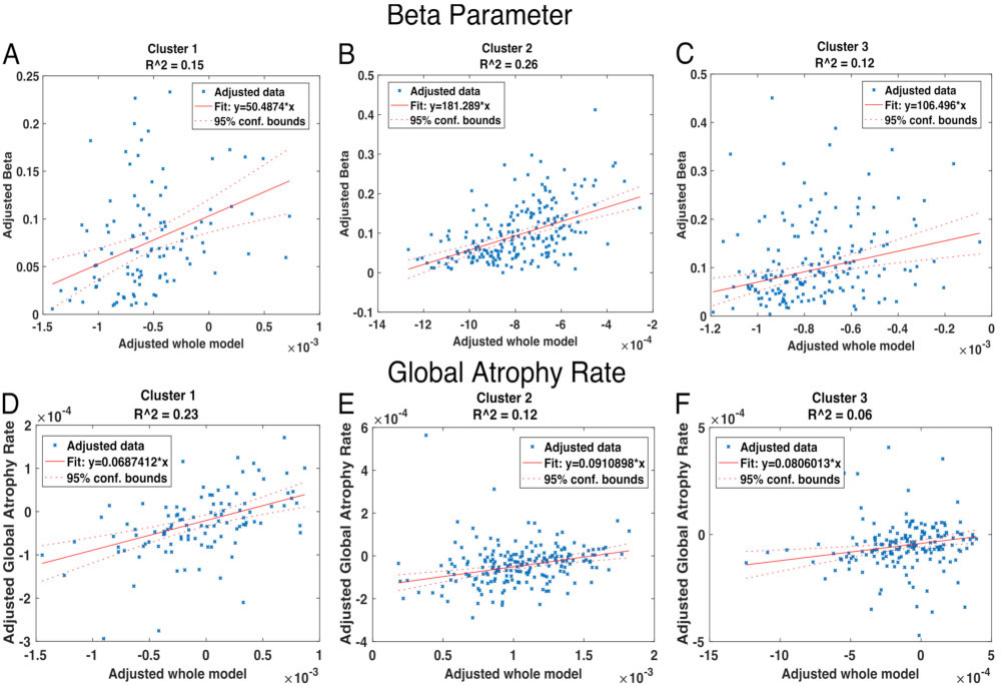
**Thirteen variable multivariable linear regressions predicting beta by cluster.** Regressions include the top 10 most atrophied regions characteristic of Alzheimer’s disease pathology and 3 CSF metrics. (**A**) To predict beta in Cluster 1 (**B**) To predict beta in Cluster 2 (**C**) To predict beta in Cluster 3 (**D**) To predict global atrophy rate in Cluster 1 (**E)** To predict global atrophy rate in Cluster 2 (**F**) To predict global atrophy rate in Cluster 3.

Although we were able to predict a narrow range of β based on cluster assignment from baseline CSF biomarker profiles, we wondered whether adding neuroimaging data will further improve the individual-level prediction of β. Hence, we created a regression model using the top 10 most atrophied regions, obtained by independent *t*-tests (Alzheimer’s disease versus CON): bilateral hippocampus, amygdala, entorhinal cortex, inferior temporal and middle temporal gyrus. To these were added the baseline CSF biomarkers (AB, tau and pTau) to form a 13 variable multivariate linear regression for predicting β which was the outcome variable (Fig. 8A–C). We found that the least pathologically severe cluster, Cluster 1, gives a relatively weak *R*^2^ of 0.15, the middle cluster yields a moderately strong *R*^2^ of 0.26 and the most pathologically severe Cluster 3 yields a relatively weak *R*^2^ of 0.12. In Cluster 2, the significant predictors were: CSF AB (*P* = 0.0006), right amygdala (*P* = 0.005), right hippocampus (*P* = 0.002) and left hippocampus volume (*P* = 0.01).

Next, we conducted the same regression analysis using global atrophy rate as the response variable (Fig. 8D–F). Unlike the above β regression results, the prediction of global atrophy rate using the same regressors yields the strongest *R*^2^ in the least pathologically severe Cluster 1. Cluster 2 and Cluster 3 yield relatively poor predictive ability of global atrophy rate with *R*^2^ = 0.12 and *R*^2^ = 0.06, respectively.

## Discussion

This study applies graph theoretic approaches and machine learning in a multimodal dataset of 810 subjects to obtain a new measure of PPR. Using our group’s previously published NDM of Alzheimer’s disease spread,^14^^,^^25^ we derive a single neurodegeneration rate, given by the model’s diffusivity parameter β, from each subject’s longitudinal MRI data. This rate measure depends on various natural stratifications of the Alzheimer’s disease cohorts. At the group level it is highest for Alzheimer’s disease patients, lower for LMCI and lowest for EMCI, indicating that the degenerative process speeds up over time. The rate is higher for subjects with 2 putative APOE4 alleles compared to subjects with no putative APOE4 alleles. However, these group differences mask substantial individual variability, which is shown in cluster analysis based on baseline CSF metrics. We demonstrate that 3 separate clusters emerge, which are characterized by differing degrees of Alzheimer’s disease-related pathology. These clusters clearly reflect differences in cognitive scores, APOE allele status, PPR parameter β and baseline atrophy patterns.

We then focussed on the clinically relevant problem of predicting PPR from a subject’s baseline data only. We found that baseline CSF biomarkers, even within clusters, were not able to predict individual PPR. However, a combination of CSF and MRI biomarkers at baseline can predict a narrow range of PPR values, and this prediction is most accurate in those subjects who happen to be in the middle of their degenerative course. This is the first study to propose a rate metric derived from the known mechanism of network-centric progression in Alzheimer’s disease, and the first to successfully model a narrow range of a subject’s rate of brain degeneration from baseline CSF and MRI biomarkers.

### Characterizing the PPR rate parameter

PPR was significantly correlated with global rate of atrophy, suggesting that the former can serve as a mechanistically relevant network version of the latter (Fig. 2). Although it is not expected that the proposed rate measure will completely track the established global atrophy rate measure, it is comforting that the two are closely associated—also an expected outcome, since PPR was fitted using the regional atrophy rates in the first place. To confirm that PPR converges with current hypotheses of neurological decline, we tested its effect size across diagnostic groups and APOE allele status (Fig. 2). Alzheimer’s disease subjects had the highest PPR followed by LMCI then EMCI subjects. PPR was moderately correlated with cognitive and clinical decline as measured by ΔMMSE, a stronger association than that between ΔMMSE and global slope of atrophy (Fig. 2C–E), supporting the enhanced role of PPR as a measure of progression in Alzheimer’s disease.

These results are consistent with the prion hypothesis of Alzheimer’s disease, whereby more advanced stages will have higher rates of progression.^12^^,^^13^^,^^41^ However, the middle stages, especially LMCI, do not have the highest rate—this is at variance to Jack’s model which involves a biomarker plateau at advanced stages.^1^^,^^42^ It is possible that empirical measurements of tau PET directly will resolve this discrepancy, but at this time sufficient longitudinal tau PET data are not available to test this.

The highest PPR was seen in APOE E4/E4 subjects compared to E4/E3 and E3/E3, consistent with prior work, as the effect of APOE and amyloid beta burden on accelerated cognitive and clinical decline in both Alzheimer’s disease and cognitively normal subjects is well-established.^6^^,^^43^^,^^44^ These results robustly characterize the proposed rate measure and demonstrate that it generally agrees with current literature and hypotheses.

### Predicting PPR from baseline CSF biomarkers

The ability to predict a subject’s rate of neurodegeneration from baseline CSF and MRI biomarkers will be critical to approach the ultimate goal of circumventing costly and time-consuming longitudinal scanning procedures for prognostic applications. The baseline predictors used in this study involve CSF biomarkers and APOE rather than clinical/demographic variables, because we desire the proposed PPR to be dependent on only pathological variables to the extent possible. Simple correlation analysis revealed no significant, straightforward relationship between baseline CSF biomarker and PPR (Fig. 3). Though this result appears to reduce the direct prognostic value of our proposed PPR, it is not entirely surprising, as current molecular biomarker models posit the relationship between disease spread and CSF biomarker is dynamic and changes with stage of disease,^1^^,^^42^ suggesting more sophisticated modelling is necessary. Indeed, while we did not observe a plateau effect on β based on *clinical* stage (EMCI, LMCI, Alzheimer’s disease), we observe a strong plateau based on *pathological* stage, as discussed below.

### Machine learning to infer pathological stage

Therefore, we employed machine learning, specifically hierarchical clustering, to establish a relationship between a subject’s 6 baseline CSF biomarkers and PPR (Fig. 4). This revealed 3 distinct clusters roughly arranged in order of pathological severity, going from Cluster 1 (highest Ab42, lowest tau) to Cluster 3 (lowest Ab42, highest tau). Although there is no one-to-one mapping between CSF clusters and Alzheimer’s disease stage, we found that Cluster 1 was most enriched with EMCI and Cluster 3 by Alzheimer’s disease, suggesting a clear order of severity, both pathologically and clinically. These results are consistent with previous work, particularly,^40^^,^^45^ which demonstrated existence of similar clusters from non-ADNI Alzheimer’s disease patients. The 3 CSF-derived clusters are also arranged in order of increasing atrophy burden and display a distinct and narrow range of PPR (Fig. 5). Notably, the more pathogenic clusters also show higher PPR, cognitive impairments and frequency of putative APOE alleles (Figs. 4–6). Within clusters, patients have differing relationships to cognitive metrics (Fig. 7).

However, it is clear that CSF biomarkers alone are insufficient to correctly estimate an individual patient’s rate parameter. Partly this reflects the well-known heterogeneity amongst Alzheimer’s disease cohorts.^40^^,^^45^ Visual inspection of the 3 CSF-based clusters reveals a mix of Alzheimer’s disease, LMCI and EMCI (Fig. 7). Prior classification work in large datasets, in particular a CSF classification study in 675 subjects showed that while CSF profile was able to discriminate between neural disorders such as depression and Alzheimer’s disease, it performed poorly at classifying across different dementias, suggested dementias have intertwined pathophysiology.^3^ Therefore, we reasoned that MRI-derived biomarkers might be necessary to further improve predictive ability.

### MRI-derived atrophy and CSF biomarkers can predict PPR

When baseline neuroimaging biomarkers were introduced along with CSF biomarkers into a linear regression model, a subject’s PPR could be predicted relatively well in Cluster 2 (*R*^2^ = 0.26), and moderately in Clusters 1 and 3 (*R*^2^ ∼0.15) (Fig. 8). At first, it would appear more plausible that Cluster 3, the most pathologically severe cluster, would yield the best linear regression results. A closer inspection revealed that Cluster 3 is highly enriched with mature Alzheimer’s disease patients, and Cluster 1 for early or mild cases (Fig. 7). Cluster 2 may therefore be considered a middle phase that is most active pathologically. Previous studies have frequently observed similar behaviour, e.g. the non-linear model of biomarker progression,^46^ whereby the rate of change is highest in the middle (inflection point) and thereby reaches a plateau reflecting resource challenged environments corresponding to mature disease.^1^^,^^42^^,^^46^ In this respect, our result showing the highest rate of progression in middle pathological stages (Cluster 2) is consistent with the literature. In contrast, Fig. 8D–F shows the highest predictive ability using these same regressors to predict global atrophy rate is highest in the least pathogenic Cluster 1 and less in pathologically advanced clusters, suggesting that global atrophy may not be as sensitive as the proposed network-constrained PPR in the critical middle stages of the disease course.

### Clinical implications

Despite remarkable progress in prognostic neuroimaging and molecular biomarkers,^6^^,^^47^ the relationship between baseline and longitudinal progression is inconsistently described,^8^^,^^48^ and overall accuracy of clinical diagnosis based on cognitive metrics is low.^9^ Dependence on cognitive/clinical score as a metric of disease progression is problematic, because clinically diagnosed MCI/Alzheimer’s disease can have non-Alzheimer’s disease aetiologies, and some cognitive controls may have preclinical Alzheimer’s disease.^10^ In line with these reports, we found moderate correlations between a subject’s speed of disease spread PPR and baseline cognitive score, which differ across clusters (Fig. 7), with Cluster 2 giving the strongest relationship between β and baseline MMSE, while Cluster 1 gave the strongest association with FAQ. The *P*-values were uncorrected for multiple comparisons, and as such these results may be considered only trending towards significance. Our goal here was not to propose the fitted beta as a predictor of cognitive decline but to show that as a new measure of pathological progression it holds information about both baseline pathology and subsequent rate of decline.

Prior work supports that baseline biomarker profiles can distinguish Alzheimer’s disease from other neurological diseases, assess risk for progression to Alzheimer’s disease or distinguish progressors from non-progressors in clinical trial environments.^3–6^ However, the majority of this work has been done using amyloid and tau PET. Unfortunately, PET scans are expensive, time consuming and not readily available in all clinical settings. While lumbar puncture is hardly less intrusive than PET, it can be done in many clinical settings and CSF samples easily shipped to central laboratories for analysis.^49^ Diagnostic criteria for Alzheimer’s disease has evolved to include molecular biomarkers.^50^ Alzheimer’s disease pathology is accompanied by decrease in CSF Ab42 and increase in CSF Tau, pTau.^51–55^

Hence, we focussed on predicting a subject’s progression rate from baseline CSF biomarkers. It was found necessary to include MRI regional atrophy, which is typically available in most patients under standard-of-care MRI. We demonstrated that PPR can be reasonably predicted from baseline CSF and MRI in a large class of subjects falling within an intermediate pathological cluster. In clinical terms, this is perhaps also the group that can most benefit from a prognostic indicator of longitudinal progression.

To our knowledge, this is the first study to propose a network-based measure of progression rate and to predict it from baseline CSF and MRI biomarkers, see, e.g. Herukka et al.^49^ Although the NDM has been extensively used in various dementias,^15–23^ previous reports did not explicitly fit for the diffusivity rate in individuals or assessed its relationship with other biomarkers as we do here. We note that rigorous individual fitting of NDM was done before, for Huntington’s disease.^22^ It is our hope that the proposed rate measure will be used in a prognostic manner as a screening tool for differential diagnosis, clinical trial inclusion and therapeutic treatments. In order to facilitate adoption, we are making the computer code available freely on our website (https://radiology.ucsf.edu/research/labs/brain-networks-lab#-) and from our GitHub repository (https://github.com/Raj-Lab-UCSF).

### Limitations

Many aspects of inter-individual variability were considered out of current scope, including cognitive and brain reserve. Many approaches exist to characterize these sources of variability, which will be explored in future work. Lack of standardization of CSF protocols to detect Ab42 and tau remains a concern, with different commercial kits utilizing their own standards, anti-bodies, ranges and cut-offs.^56^ However, methodologies regarding measurement of Aβ_1–42_, t-tau and p-tau_181_ in clinically annotated ADNI CSF samples are available to identify and control key sources of analytical variability.^5^ It was suggested that longitudinal CSF data is necessary for prognostic application as biomarkers can change linearly with time, exponentially, sigmoidally, etc.^42^^,^^57^ Our study mitigates that issue as 90% of CSF data was collected within a year of subject’s baseline scan, reducing the need for extensive modelling of biomarker dynamics.

The NDM is a first-order, linear, parsimonious model of diffusive spread that assumes that the structural connectivity network remains static and unchanged over the duration of the longitudinal analysis. This is reasonable because the observation window (2–6 years) is short compared to the course of the disease. Our use of a template connectome instead of patient-specific ones is also a limitation that warrants future exploration. However, prior explorations suggest that subject-specific connectomes are no better than template connectomes regarding the NDM.^58^ Additionally, although the model enables long-term projections of future atrophy, model validation in the current work is limited to public (ADNI) data sets of rather narrow time span (2–6 years), precluding long-term longitudinal follow-up.

## Supplementary material

Supplementary material is available at *Brain Communications* online.

## Supplementary Material

fcab144_Supplementary_Data

## Abbreviations

Aβ =: amyloid beta
ADAS-Cog: = Alzheimer’s disease assessment scale—cognitive
ADNI =: Alzheimer’s Disease Neuroimaging Initiative
APOE =: apolipoprotein-E
CON =: healthy controls;
EMCI =: Early Mild Cognitive Impairment
FAQ =: Functional Activities Questionnaire
GM =: grey matter
LMCI =: Late Mild Cognitive Impairment
MMSE =: Mini-Mental State Exam
NDM =: Network Diffusion Model
PPR: Pathology progression rate
pTau =: phosphorylated tau
WM: white matter
