# Supplementary material for: Network-constrained technique to characterize pathology progression rate in Alzheimer’s disease
Source: Brain Commun. 2021 Jul 15;3(3):fcab144. doi: 10.1093/braincomms/fcab144 (PMC8376686; doi:10.1093/braincomms/fcab144)
Supplement: fcab144_Supplementary_Data [file fcab144_supplementary_data.pdf]

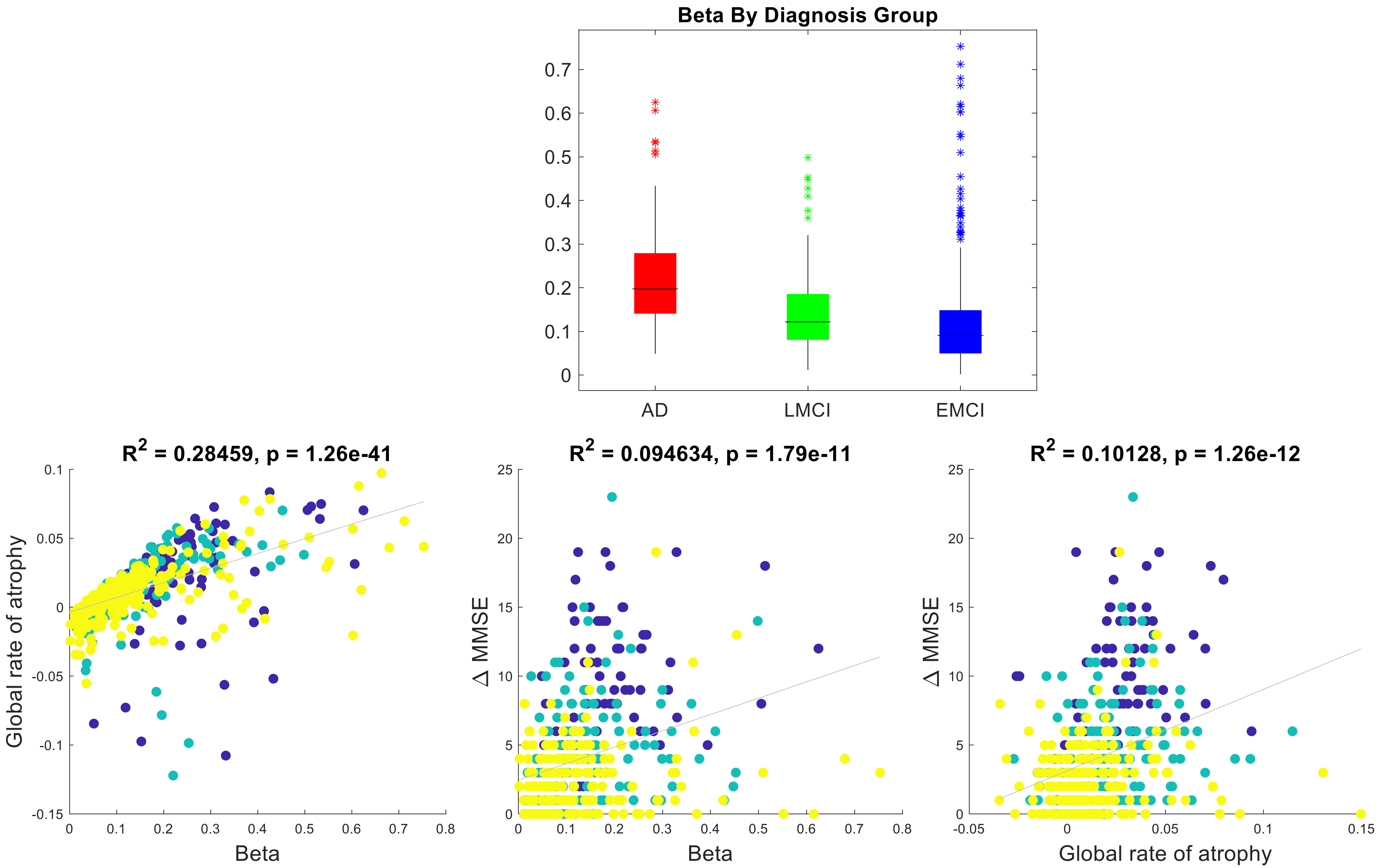

Supplementary Figure S1, analogous to **Figure 2**. Reproducibility of significant effect of PPR on subject's atrophy rate, MMSE and diagnosis, using a different, more modern connectome from the Human Connectome Project.
